# Association of Roadway Proximity with Fasting Plasma Glucose and Metabolic Risk Factors for Cardiovascular Disease in a Cross-Sectional Study of Cardiac Catheterization Patients

**DOI:** 10.1289/ehp.1306980

**Published:** 2015-03-24

**Authors:** Cavin K. Ward-Caviness, William E. Kraus, Colette Blach, Carol S. Haynes, Elaine Dowdy, Marie Lynn Miranda, Robert B. Devlin, David Diaz-Sanchez, Wayne E. Cascio, Shaibal Mukerjee, Casson Stallings, Luther A. Smith, Simon G. Gregory, Svati H. Shah, Elizabeth R. Hauser, Lucas M. Neas

**Affiliations:** 1 Duke Molecular Physiology Institute, and; 2 Division of Cardiovascular Medicine, Duke University Medical Center, Durham, North Carolina, USA; 3 School of Natural Resources and the Environment, University of Michigan, Ann Arbor, Michigan, USA; 4 National Health and Environmental Effects Research Laboratory, U.S. Environmental Protection Agency (EPA), Chapel Hill, North Carolina, USA; 5 National Exposure Research Laboratory, U.S. EPA, Research Triangle Park, North Carolina, USA; 6 Alion Science and Technology Inc., Durham, North Carolina, USA; 7 Epidemiologic Research and Information Center, Durham Veterans Affairs Medical Center, Durham, North Carolina, USA

## Abstract

**Background:**

The relationship between traffic-related air pollution (TRAP) and risk factors for cardiovascular disease needs to be better understood in order to address the adverse impact of air pollution on human health.

**Objective:**

We examined associations between roadway proximity and traffic exposure zones, as markers of TRAP exposure, and metabolic biomarkers for cardiovascular disease risk in a cohort of patients undergoing cardiac catheterization.

**Methods:**

We performed a cross-sectional study of 2,124 individuals residing in North Carolina (USA). Roadway proximity was assessed via distance to primary and secondary roadways, and we used residence in traffic exposure zones (TEZs) as a proxy for TRAP. Two categories of metabolic outcomes were studied: measures associated with glucose control, and measures associated with lipid metabolism. Statistical models were adjusted for race, sex, smoking, body mass index, and socioeconomic status (SES).

**Results:**

An interquartile-range (990 m) decrease in distance to roadways was associated with higher fasting plasma glucose (β = 2.17 mg/dL; 95% CI: –0.24, 4.59), and the association appeared to be limited to women (β = 5.16 mg/dL; 95% CI: 1.48, 8.84 compared with β = 0.14 mg/dL; 95% CI: –3.04, 3.33 in men). Residence in TEZ 5 (high-speed traffic) and TEZ 6 (stop-and-go traffic), the two traffic zones assumed to have the highest levels of TRAP, was positively associated with high-density lipoprotein cholesterol (HDL-C; β = 8.36; 95% CI: –0.15, 16.9 and β = 5.98; 95% CI: –3.96, 15.9, for TEZ 5 and 6, respectively).

**Conclusion:**

Proxy measures of TRAP exposure were associated with intermediate metabolic traits associated with cardiovascular disease, including fasting plasma glucose and possibly HDL-C.

**Citation:**

Ward-Caviness CK, Kraus WE, Blach C, Haynes CS, Dowdy E, Miranda ML, Devlin RB, Diaz-Sanchez D, Cascio WE, Mukerjee S, Stallings C, Smith LA, Gregory SG, Shah SH, Hauser ER, Neas LM. 2015. Association of roadway proximity with fasting plasma glucose and metabolic risk factors for cardiovascular disease in a cross-sectional study of cardiac catheterization patients. Environ Health Perspect 123:1007–1014; http://dx.doi.org/10.1289/ehp.1306980

## Introduction

Cardiovascular diseases (CVDs) are the primary cause of death in developed nations (Lopez et al. 2006). Metabolic risk factors such as high-density lipoprotein cholesterol (HDL-C) and total cholesterol (TC) are often an important component of multivariate CVD risk prediction models (D’Agostino et al. 2001; Kannel et al. 1976; Pencina et al. 2009). Other metabolic risk factors may not appear in risk prediction models but remain strong risk factors for CVD, such as diabetes mellitus, fasting plasma glucose (FPG), insulin resistance (homeostatic model assessment method–insulin resistance; HOMA-IR), low-density lipoprotein cholesterol (LDL-C), and triglycerides (TG). These metabolic risk factors may be mechanistically linked to cardiovascular disease (Ginsberg 2000), are potentially modifiable, and may be affected by environmental factors such as air pollution (Chuang et al. 2010, 2011; Thiering et al. 2013).

Air pollution is an independent risk factor for cardiovascular disease (Brook et al. 2010), and specific sources and components may be linked to cardiovascular disease (Peng et al. 2009; Zanobetti et al. 2009). Urban and traffic-related air pollution have been associated with coronary atherosclerosis and cardiovascular events (Hoffmann et al. 2006, 2007, 2009) as well as multiple metabolic risk factors for CVD, including diabetes (Brook et al. 2008; Krämer et al. 2010; Pearson et al. 2010; Peters 2012), LDL-C (Kelishadi et al. 2009), FPG, and HDL-C (Chuang et al. 2010). These metabolic risk factors can be grouped into two categories: those related to glucose control and those related to lipid metabolism. Measures of glucose control are linked with CVD, with a 1-unit increase in insulin resistance associated with a 5.4% increased risk of CVD (Reddy et al. 2010). Lipids and their metabolism, particularly LDL-C, may play a mechanistic role in the pathogenesis of cardiovascular disease (Tabas 2011). HDL-C is thought to be protective against CVD, and high levels of blood cholesterol and triglycerides are considered CVD risk factors (Assmann and Gotto 2004). Serum lipids are influenced by diet (Mensink and Katan 1992). In addition, exposure to particulate matter air pollution has been associated with markers of oxidative damage to serum lipids (Møller and Loft 2010).

The CATHeterization GENetics (CATHGEN) cohort is a large cardiac catheterization cohort with a single sampling site, Duke University Medical Center. As such, 25% of the cohort comes from Durham, Wake, and Orange counties in North Carolina (NC). These are three of the most urban counties in NC, containing the major cities of Durham, Raleigh, and Chapel Hill, respectively. In addition, studies have shown that particulate air pollution in Raleigh is correlated with distance to major roadways (Hagler et al. 2009). Utilizing CATHGEN participants from this tri-county area, we seek to better understand the relationship between traffic-related air pollution (TRAP) and metabolic risk factors for CVD.

## Methods

*Study population*. All individuals in this study came from the CATHGEN cohort, a large cardiovascular cohort of 9,334 adult patients recruited at the Duke University Cardiac Catheterization Clinic from January 2001 through December 2010. At the time of catheterization, a medical fellow or attending physician administered an intake Health and Physical (HP) examination, which covered clinical conditions and risk factors. Blood and serum samples were collected during study enrollment, and the patient’s medical records associated with the catheterization were merged into our secure database. We obtained patient addresses from billing records. All participants received and signed informed consent forms prior to enrollment, and the CATHGEN biorepository has been approved by and follows all Duke University Institutional Review Board policies (Shah et al. 2010b). None of the samples in CATHGEN were collected from catheterizations performed in the context of an acute coronary syndrome because informed consent could not be obtained in these cases.

**Figure 1:**
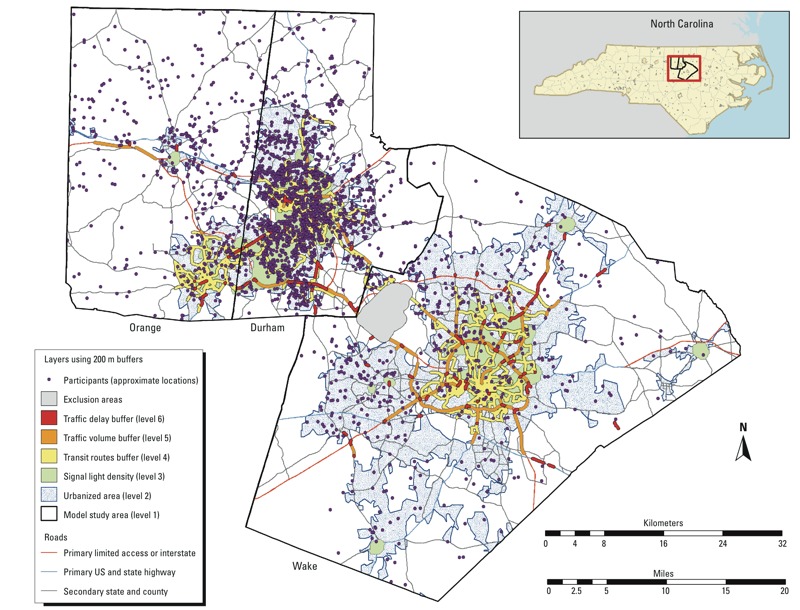
CATHGEN participants in study area. The location of the study area in the state of North Carolina (top right), and the distribution of CATHGEN participants within the study area of Durham, Wake, and Orange counties. The participant locations are overlaid on a map of the primary and secondary roadway network, as well as the traffic exposure zones; the participant locations have been randomized on a small scale to protect confidential patient information while preserving the overall spatial distribution.

The Children’s Environmental Health Initiative (http://cehi.snre.umich.edu/) mapped residential addresses to geocoded latitude and longitude for the present study. Individuals were considered successfully geocoded at the street segment level. Of 9,334 individuals enrolled in CATHGEN during the study period, 8,017 (86%) were successfully geocoded, 7,118 (76%) resided in North Carolina, and 2,318 (25%) resided in our study area of Durham, Wake, and Orange counties (Figure 1). For participants whose addresses changed over time, we used the most recent address that was entered into their record before the catheterization. The average time at an address prior to the catheterization procedure according to our records was 587 days.

We excluded 64 participants who did not self-report as European American (EA) or African American (AA). We used ArcGIS (release 10; ESRI 2011) along with roadway information from the North Carolina Department of Transportation to calculate the perpendicular distance from each participant’s address at the time of catheterization to the nearest primary or secondary roadway, and excluded 130 participants (5.8% of the cohort) who resided > 2 mi (3.22 km) from a primary or secondary roadway, leaving a study cohort of 2,124 individuals for this analysis (Table 1).

**Table 1:** Demographic and clinical variables for the study cohort and race/sex stratified subcohorts used for analysis.

Covariate	All	Male	Female	AA	EA
*n*	2,124	1,274	850	625	1,499
Sex (% female)	40.0	0.0	100.0	50.4	35.7
Race (% EA)	70.5	75.6	62.9	0.0	100.0
Age (years)	61.3 ± 12.2	60.7 ± 11.9	62.3 ± 12.6	57.4 ± 11.6	63.0 ± 12.0
BMI (kg/m^2^)	30.3 ± 7.36	29.9 ± 6.60	31.0 ± 8.33	32.6 ± 8.24	29.4 ± 6.75
Ejection fraction (%)	57.9 ± 12.8	56.4 ± 13.0	60.0 ± 12.3	56.3 ± 13.7	58.5 ± 12.4
FPG (mg/dL)	117 ± 48.1	119 ± 49.8	114 ± 45.3	124 ± 58.4	114 ± 42.7
Total cholesterol (mg/dL)	183 ± 47.5	179 ± 46.9	190 ± 47.8	185 ± 48.7	182 ± 47.0
Triglycerides (mg/dL)	166 ± 159	178 ± 187	146 ± 97.2	133 ± 85.8	180 ± 181
LDL-C (mg/dL)	104 ± 40.3	102 ± 38.7	107 ± 42.7	108 ± 41.8	102 ± 39.5
HDL-C (mg/dL)	47.5 ± 17.1	43.7 ± 14.7	53.6 ± 18.9	49.1 ± 17.5	46.7 ± 16.8
Hyperlipidemia	60.5	64.1	55.2	57.1	62.0
Hypertension	69.3	67.9	71.4	81.3	64.3
CAD	41.7	50.3	28.8	31.4	46.0
Diabetes (HP)	28.5	29.8	26.6	40.0	23.7
Smoking status (% smokers)	44.6	50.0	36.5	43.0	45.2
HOMA-IR	3.97 ± 5.84	3.93 ± 6.35	4.01 ± 5.08	5.13 ± 7.88	3.67 ± 4.34
SES, median home value ($1,000s)	179 ± 93.8	185 ± 94.2	171 ± 92.5	129 ± 68.6	200 ± 95.0
Distance to roadways (m)	898 ± 747	918 ± 759	868 ± 729	784 ± 663	946 ± 775
Distance to roadways IQR (m)	990	1,031	929	895	1,020
TEZ 1	19.2	20.3	17.4	10.1	23.0
TEZ 2	36.0	38.3	32.6	24.0	41.0
TEZ 3	15.0	13.7	16.8	15.2	14.9
TEZ 4	27.4	25.1	30.7	47.0	19.2
TEZ 5	1.5	1.4	1.7	2.4	1.1
TEZ 6	1.0	1.1	0.8	1.3	0.9
Abbreviations: AA, African Americans; BMI, body mass index; CAD, coronary artery disease; EA, European Americans; FPG, fasting plasma glucose; HDL-C, high-density lipoprotein cholesterol; HOMA-IR, homeostatic model assessment method–insulin resistance; HP, health and physical examination; IQR, interquartile range; LDL-C, low-density lipoprotein cholesterol; SES, socioeconomic status; TEZ, traffic exposure zone. Values shown are mean ± SD for continuous variables and percentage for categorical variables. SES was assessed using the median home value at the census-block level.					

*Clinical information*. Clinical information used in this study came from the HP exam at the time of the catheterization, medical records associated with the catheterization, and measurements made using the stored patient blood and plasma samples. Based on available records, approximately 82% of individuals approached to be in the CATHGEN biorepository consented, and no patients were excluded due to age, race, or sex. The CADindex is an ordinal measure that indexes the future probability of a future adverse cardiovascular event based on measurements of coronary atherosclerosis made during the cardiac catheterization procedure (Wang et al. 2007). By defining coronary artery disease (CAD) as a CADindex > 23 (Wang et al. 2007), we determined that 42% of our population has CAD. All of the clinical variables, including associated laboratory measures for FPG, LDL-C, HDL-C, TG, and total cholesterol (TC), were collected at the time of catheterization. The HP exam was a standard exam provided to all patients, with no additional questions based on the individual’s consent to participate in CATHGEN. All blood draws for lab measures, including FGP, were done immediately prior to the catheterization procedure, thus there was no delay between collection of data based on the HP exam and lab values. We used two binary measures of diabetes. One measure was defined as an indication of either type I or type II diabetes on the HP exam. This HP exam annotation would have been made by the attending physician administering the exam, and we refer to this measure as “diabetes (HP).” The second measure was based on the patient’s FPG, with diabetes being defined as a fasting plasma glucose ≥ 126 mg/dL (American Diabetes Association 2008). We refer to this measure as “diabetes (FPG).” There was strong concordance between these two measures, with 80% (1,709/2,124) of individuals concordant diabetes status according to the two measures: 1,373 positive and 336 negative. There were 145 diabetes (FPG) positive individuals not recorded as having diabetes according to the diabetes (HP), and 270 individuals with a positive indication for diabetes (HP) did not have diabetes as determined by their FPG. We additionally performed stratified analyses restricting to diabetic individuals (as determined by FPG) because these individuals may already have impaired glucose metabolism and thus be susceptible to further metabolic dysfunction as a result of air pollution exposure. Also to align with previous studies that mostly centered on type II diabetes, we performed logistic regression analyses restricting diabetes cases to individuals with reported type II diabetes according to their HP exam (*n* = 573). Insulin was measured via mass-spectroscopy on a blood sample taken during the catheterization (Shah et al. 2010a), and insulin resistance (HOMA-IR) was calculated via the homeostatic model assessment method (Levy et al. 1998; Matthews et al. 1985), as (insulin * FPG)/405. Hyperlipidemia was defined as a previous diagnosis or treatment for hypercholesterolemia based on information taken from the medical record. We separated the outcomes into those related to glucose control (diabetes, FPG, HOMA-IR) and those related to lipid metabolism (LDL-C, HDL-C, TG, TC, hyperlipidemia). Correlations between pairs of continuous outcomes that include one glucose control outcome and one lipid metabolism outcome ranged from –0.15 (for HOMA-IR and HDL-C) to 0.31 (for HOMA-IR and TG) (see Supplemental Material, Table S1).

Models incorporating these outcomes were adjusted for race (AA or EA), sex, body mass index (BMI; based on height and weight at the HP examination), smoking status (positive or negative), and median home value in the participant’s census block (as a proxy for SES). Smoking status was defined by the Duke Institute for Clinical Cardiovascular Care as positive if participants smoked ≥ 10 cigarettes/day currently or had quit smoking ≥ 10 cigarettes/day within the past 5 years because of their cardiovascular disease, or as negative otherwise. Median home value was determined using 2000 Census data for patients enrolled before 2 July 2005 (49.7% of participants) and using 2010 census data otherwise.

*Indexing of traffic-related air pollution*. We used the 2010 TIGER/Line® Shapefiles (Topologically Integrated Geographic Encoding and Referencing) North Carolina Primary and Secondary Roads State-based Shapefile (4th quarter release) as our reference data set for the North Carolina roadway network (https://connect.ncdot.gov/resources/gis/Pages/GIS-Data-Layers.aspx). This file is compatible with the ArcGIS® software suite (ESRI 2011) and was in the same geospatial reference frame as the geocoding performed by the Children’s Environmental Health Initiative. The roadway network used for analysis was updated to the most current version (2010 4th quarter release) as of the initiation of analysis to incorporate the most precise exposure information. Primary and secondary roads were classified by the North Carolina Department of Transportation (NCDOT 2015) using criteria consistent with the Master Address File/Topologically Integrated Geographic Encoding and Referencing Feature Class Code definitions used by the U.S. Census Bureau (2013). Specifically, primary roads are divided limited-access highways with interchanges, and secondary roads are main inter- and intra-city arteries with multiple lanes of traffic that usually have at-grade intersections with other roads and driveways, including those of residential housing.

We used the inverse natural logarithm of the minimum perpendicular distance from the participant’s residence to a primary or secondary roadway as one of our indices of TRAP. The inverse logarithm transform was applied because it reflects the functional form of the relation between distance to roadways and the concentration of particulate air pollution (Hagler et al. 2009). Exploratory analysis of associations between the outcomes and roadway proximity modeled using thin-plate smoothing splines implemented in the mgcv package (Wood 2003) in the R statistical programming language (R Core Team 2009) confirmed that the inverse logarithm transform linearized the exposure–response relationship (for the association with FPG, see Supplemental Material, Figure S1).

*Traffic exposure zones*. In a study of wheezing in infants, geographic zones defined by traffic types yielded positive associations (Ryan et al. 2005). For Durham, Wake, and Orange counties, we used six mutually exclusive but noncontiguous traffic exposure zones (TEZs), which categorize exposure to traffic-related air pollution as described by Mukerjee et al. (2015). The TEZs utilized were high-volume traffic with regular congestion-related delays (TEZ 6), high-volume traffic with smooth flow (TEZ 5), mass transit routes (TEZ 4), high-traffic urbanized areas with signal light density (TEZ 3), urbanized areas (TEZ 2), and the remainder of the study area (TEZ 1). A 1-km buffer around the Raleigh–Durham airport was excluded from the study.

As detailed by Mukerjee et al. (2015), TEZs 4, 5, and 6 were constructed using traffic model data supplied by the Institute for Transportation Research and Education of North Carolina State University (http://www.itre.ncsu.edu/). TEZ 6 included areas within 200 m of road segments with high-volume traffic (> 40,000 vehicles/day) and congestion-related delays of ≥ 35% during morning peak traffic (as estimated on a typical day in the fall or spring of 2005). TEZ 5 included areas within 200 m of road segments with high-volume traffic and smooth flow, and TEZ 4 included areas within 200 m of transit routes for the cities of Chapel Hill, Raleigh, and Durham, including transit routes for the University of North Carolina at Chapel Hill and North Carolina State University in Raleigh. TEZ 3 included areas with > 3 traffic lights per mile (high signal light–density zones). TEZ 2 included urbanized areas, as defined by the 2000 Census, and all areas not included in one of the other zones were classified as TEZ 1, the reference zone for all analyses. The final map of TEZs was created by first defining the study area (set as TEZ 1 by default), then overlaying urbanized areas (TEZ 2), then all areas classified as having high signal light density (TEZ 3), and so on through TEZ 6. CATHGEN participants were classified according to the highest zone that contained their geocoded address (Figure 1).

*Statistical methods*. We used R v2.10.1 (R Core Team 2009) for all analyses. Generalized additive models, as implemented in the mgcv package (Wood 2008), were used to estimate associations of our two TRAP proxies with metabolic outcomes. Generalized additive models allow for more complete model diagnostics than traditional linear models. Also, because linear models are a special subset of generalized additive models, generalized additive models are appropriate even in the event that no smoothed relationships are necessary.

The outcomes analyzed for association with our proxies for TRAP were FPG, diabetes (HP), diabetes (FPG), HOMA-IR, hyperlipidemia, LDL-C, HDL-C, TC, and TG. The risks of the binary outcomes [diabetes (HP), diabetes (FPG), and hyperlipidemia] were estimated using a logistic model with a quasi-binomial transformation. The quasi-binomial transformation allows for overdispersion and can thus provide more accurate and stable model estimates.

All models were adjusted for race (AA or EA), sex, BMI (continuous), smoking status (positive or negative, as previously defined), and median home value (census-block level, continuous). We did not adjust for age at catheterization because it is a potential causal intermediate between TRAP exposure and the outcomes of interest; cardiovascular effects of TRAP may reduce the age at which CATHGEN participants present for catheterization and are enrolled into the cohort. Similarly, we did not adjust for clinical variables such as hypertension and extent of cardiovascular disease because they also may be causal intermediates between TRAP exposure and the metabolic outcomes.

Associations between TRAP exposure and the outcomes were modeled using two proxy measures of exposure: distance from the participant’s residence to the nearest primary or secondary roadway, and the traffic exposure zone of each participant’s residence. For the distance to primary or secondary roadways exposure, the inverse-logarithm transform was scaled to the interquartile range (IQR; 990 m), and results are expressed in terms of the regression coefficient (β) for continuous outcomes or the odds ratio (OR) for binary outcomes. Because the βs and ORs are presented on the scale of an IQR increase in the transformed distance to roadway variable, they represent a proportional change on the untransformed scale.

Models to estimate associations with the TEZs included five indicator variables for the six TEZ areas, with TEZ 1 used as the reference group for all comparisons. Given the small sample sizes in TEZs 5 and 6, they were combined into one TEZ (TEZ 5/6) in a secondary analysis for all outcomes. To test for an association with a trend across the traffic exposure zones, a linear association with an ordinal TEZ variable was used. As with models of associations with distance to roadways, TEZ models were adjusted for race, sex, BMI, smoking status, and median census block home value. Again, given their small sample sizes, TEZ 5 and TEZ 6 were combined into a single TEZ (TEZ 5/6) for some analyses. For the TEZ exposure, results are expressed in terms of the regression coefficient for continuous outcomes or odds ratio for binary outcomes.

All associations were examined for influential observations using Cook’s distance (Cook 1977), and any observations found to be exerting undue influence were removed. After examination, only a single observation was removed for the outcome HOMA-IR. In addition to modeling associations among all participants combined, we estimated associations separately for men and women, and for AA and EA, using stratified models.

## Results

*Observed characteristics of study population*. The study population consisted of EA and AA CATHGEN participants residing 2 mi (3.22 km) from a primary or secondary roadway in Durham, Wake, and Orange counties (Table 1). The IQR for distance to a primary or secondary roadway among the study population was 990 m. Our study population was 40% female, with an average age of 61 years and BMI of 30 kg/m^2^. The study population was concentrated in TEZs 1–4, with most living in TEZ 2 (urbanized areas) (*n* = 765).

**Figure 2:**
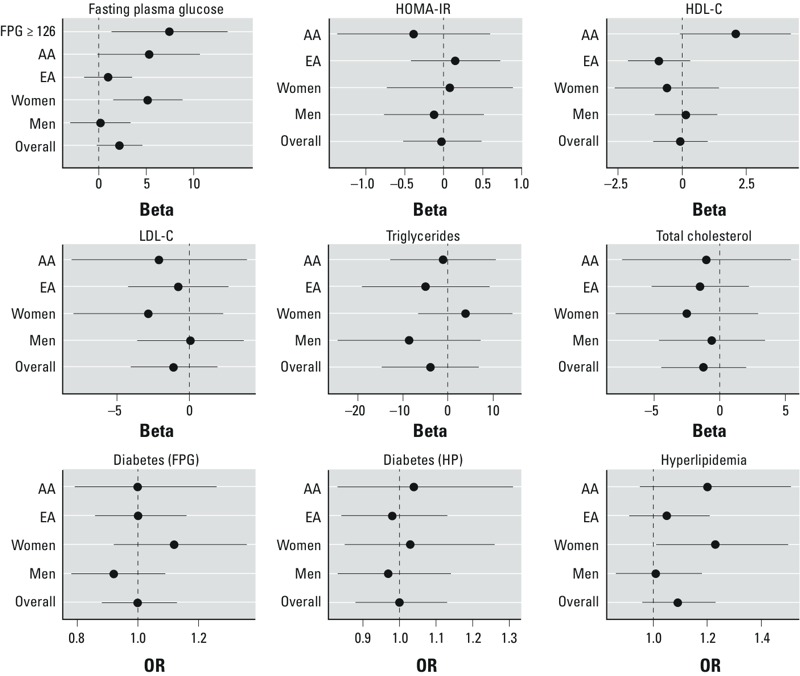
Association of glucose control and lipid metabolism outcomes with distance to roadways. Abbreviations: AA, African Americans; BMI, body mass index; EA, European Americans; FPG, fasting plasma glucose; HDL‑C, high-density lipoprotein cholesterol; HOMA‑IR, homeostatic model assessment method–insulin resistance; HP, health and physical examination; LDL‑C, low-density lipoprotein cholesterol. Forest plots of the association between distance to roadways and metabolic outcomes. Associations are presented as an effect estimate (Beta) for the continuous outcomes and as an OR for the binary outcomes. Associations were scaled so that a 1-unit change corresponds to the IQR (990 m); error bars indicate 95% CIs. Models were adjusted for race, sex, smoking status, socioeconomic status (median house value at census-block level), and BMI. Complete numeric data are provided in Supplemental Material, Table S2.

*Glucose control and diabetes results*. An IQR decrease in the inverse-logarithm transform of distance to roadways was associated with an increase in FPG concentrations [β = 2.17 mg/dL; 95% confidence interval (CI): –0.24, 4.59; *p* = 0.078] (Figure 2; see also Supplemental Material Table S2). This association was stronger among those with FPG ≥ 126 mg/dL (β = 7.45 mg/dL; 95% CI: 1.30, 13.6; *p* = 0.018), among AA (β = 5.28 mg/dL; 95% CI: –0.17, 10.7; *p* = 0.058) compared with EA (β = 0.96 mg/dL; 95% CI: –1.61, 3.52; *p* = 0.47), and among women (β = 5.16 mg/dL; 95% CI: 1.48, 8.84; *p* = 0.006) compared with men (β = 0.14 mg/dL; 95% CI: –3.04, 3.33; *p* = 0.930).

**Figure 3:**
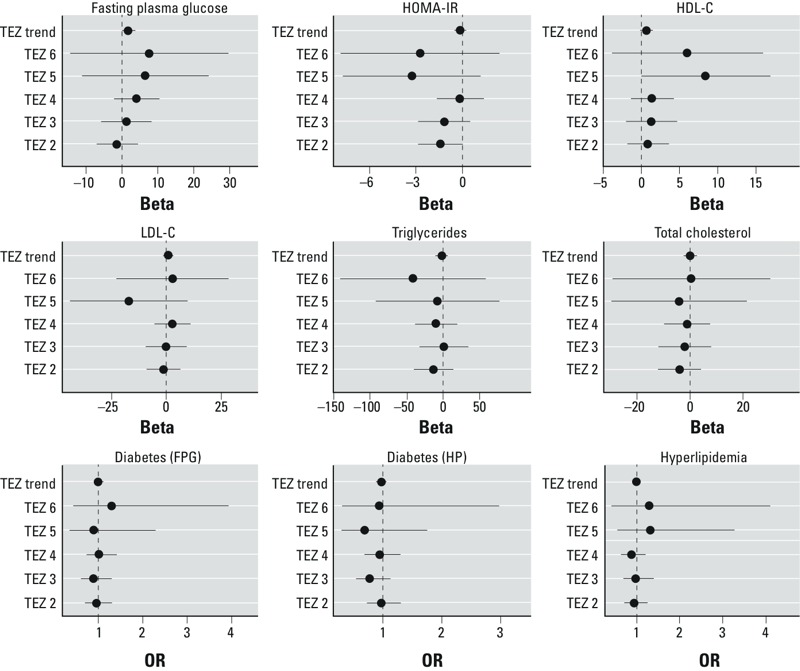
Association of glucose control and lipid metabolism outcomes with traffic exposure zones. Abbreviations: BMI, body mass index; FPG, fasting plasma glucose; HDL‑C, high-density lipoprotein cholesterol; HOMA‑IR, homeostatic model assessment method–insulin resistance; HP, health and physical examination; LDL‑C, low-density lipoprotein cholesterol; TEZ, traffic exposure zone. Forest plots show the association between TEZ and metabolic outcomes. Associations are presented as an effect estimate (Beta) for the continuous outcomes and as an OR for the binary outcomes. Associations with individual TEZs are presented as compared with the baseline zone (TEZ 1). For the TEZ trend association, the TEZs were treated as a single ordinal variable. Error bars indicate 95% CIs. Models were adjusted for race, sex, smoking status, socioeconomic status (median home value at census-block level), and BMI. Complete numeric data are provided in Supplemental Material, Table S3.

None of the individual TEZs were associated with FPG (Figure 3; see also Supplemental Material Table S3). However, associations increased monotonically as the zones increased from TEZ 3 to TEZ 6 (trend *p*-value = 0.07, Figure 3).

We did not observe an association between diabetes (HP), diabetes (FPG), or HOMA-IR and our TRAP exposure metrics (Figures 2 and 3; see also Supplemental Material, Tables S2 and S3). We additionally restricted our diabetes (HP) measure to those individuals diagnosed with type II diabetes (*n* = 573), but still did not note any associations (*p* < 0.05) with TRAP (data not shown).

*Lipid metabolism results*. Decreasing distance to roadways was not associated with hyperlipidemia in the overall cohort (OR = 1.09; 95% CI: 0.96, 1.23; *p* = 0.17) (Figure 2; see also Supplemental Material, Table S2). Distance to roadways was associated with hyperlipidemia among women (OR = 1.23; 95% CI: 1.01, 1.50; *p* = 0.042) but not men (OR = 1.01; 95% CI: 0.86, 1.18; *p* = 0.94).

HDL-C was not associated with distance to roadways in the overall population, but associations indicated higher HDL-C concentrations among AA as the distance to roadways decreased (β = 2.08 mg/dL; 95% CI: –0.09, 4.24; *p* = 0.06) compared with EA (β = –0.09; 95% CI: –2.13, 0.31). Residence in TEZ 5 was positively associated with HDL-C (β = 8.36 mg/dL compared with TEZ 1; 95% CI: –0.15, 16.9; *p* = 0.054) (Figure 3; see also Supplemental Material, Table S3). In the analysis of TEZ 5/6, the association remained positive while the associated confidence interval shrank (β = 7.36 mg/dL; 95% CI: 0.74, 14.0, *p* = 0.03).

Neither distance to roadway nor the TEZs were associated with LDL-C, TG, or TC (Figures 2 and 3; see also Supplemental Material, Tables S2 and S3.)

## Discussion

In this study we explored the relations between traffic-related air pollution and two groups of metabolic risk factors for CVD: those related to glucose control (FPG, HOMA-IR, and diabetes), and those related to lipid metabolism (LDL-C, HDL-C, TG, TC, and hyperlipidemia). We used two separate indices for traffic-related air pollution: *a*) the inverse-natural logarithm of the distance to the nearest primary or secondary roadway (scaled to the IQR) and *b*) TEZs. Decreasing distance to roadways was positively associated with FPG (β = 2.17 mg/dL; 95% CI: –0.25, 4.58). Air pollution has been previously associated with FPG (Brook et al. 2008; Chuang et al. 2011; Krämer et al. 2010) and reported to be stronger among women than men (Brook et al. 2008; Krämer et al. 2010). We also observed this women-specific association in the CATHGEN cohort (β = 5.16 mg/dL; 95% CI: 1.48, 8.84).

There is currently little literature on the impact of racial/ethnic stratifications on associations between air pollution and metabolic outcomes. We found an association between FPG and an IQR decrease in distance to roadways among AA (β = 5.28 mg/dL; 95% CI: –0.17, 10.7) but not EA (β = 0.96 mg/dL; 95% CI: –1.61, 3.52). To our knowledge, this is the first report of racial differences in associations with FPG. Each of the race-specific models was adjusted for median home value at the census tract level, and this area-level adjustment for SES did not impact associations (data not shown). We do acknowledge that unaccounted for demographic variables may impact these race-specific associations, including individual-level SES metrics; thus, future studies should extend the demographic variables collected to more fully evaluate potential confounders.

Several markers of TRAP have been associated with diabetes, including nitrogen dioxide (NO_2_) (Andersen et al. 2012; Brook et al. 2008; Coogan et al. 2012), traffic-related particulate matter (Krämer et al. 2010), and distance to roadways (Puett et al. 2011). Often associations between traffic-related air pollution and diabetes are observed only among women (Hoek et al. 2013; Rajagopalan and Brook 2012). The strongest association with outcomes related to glucose control in our study population was between FPG and distance to roadways among women. We observed no strong differences in exposure or confounders that would yield insight into this sex-specific association. Although there may be an underlying biological basis for this, perhaps related to sex-specific hormone and metabolic differences, we cannot discount that the differences may be to as yet unmeasured confounders, including differences related to exposure to specific air pollution components, SES, or daily routines.

In a series of papers exploring the metabolic consequences of air pollution exposure, researchers have shown that mechanisms related to glucose control are disrupted after exposure to PM_2.5_ (particulate matter ≤ 2.5 μm in aerodynamic diameter) (Rajagopalan and Brook 2012; Xu et al. 2011; Zheng et al. 2013). Animals exposed to PM_2.5_ experience increases in insulin resistance, indicating metabolic dysfunction (Sun et al. 2009; Xu et al. 2011). A likely cause of this dysfunction is oxidative stress. Oxidative stress is known to induce dysfunction in glucose control (Rains and Jain 2011), and oxidized lipids, such as LDL, have been proposed as a biological marker for exposure to air pollution (Møller and Loft 2010). Future work should jointly examine metabolic markers and oxidative stress in human studies to connect TRAP, oxidative stress, and metabolic outcomes in a way that can evaluate potentially causal relationships among these variables.

The specificity of associations between air pollution and measures of glucose control outcomes requires further exploration. Chuang et al. (2011) investigated associations between the annual average of several air pollution measures and cardiovascular risk factors in 1,023 elderly individuals from Taiwan. They reported that FPG and hemoglobin A1c were associated with PM_2.5_ and NO_2_. The association between distance to roadways and FPG in our study population is consistent with that study. Longitudinal cohort studies, such as the Chinese Air Pollution and CardioMetabolic Disease cohort (Sun et al. 2013), that examine multiple primary and secondary cardiometabolic end points will be extremely useful in examining the specificity of associations between traffic-related air pollution and multiple cardiometabolic outcomes.

Our lipid metabolism outcomes were LDL-C, HDL-C, TG, TC, and hyperlipidemia. Air pollution has been associated with oxidized LDL-C (Kelishadi et al. 2009), but in a study of traffic exposures there was no difference in HDL-C or LDL-C levels when the population was classified into low- and high-traffic–exposure cohorts (Hoffmann et al. 2006). In our analyses, distance to roadways was associated with hyperlipidemia among women (OR = 1.23; 95% CI: 1.01, 1.50), and residence in TEZ 5/6 was associated with increased HDL-C (β = 7.36; 95% CI: 0.74, 14.0), an association that needs further exploration because HDL is often thought of as a protective factor for CVD. Again, we suggest that future studies work on jointly measuring TRAP exposure, oxidative stress markers, and metabolic outcomes in order to establish causal relationships between these variables.

The aforementioned study of cardiovascular risk factors among the elderly in Taiwan (Chuang et al. 2011) analyzed the lipid metabolism–related outcomes HDL-C, TC, and TG. Similar to our associations, they noted specific associations between these outcomes and long-term air pollution exposure. However, in their analyses only TC showed associations with PM_2.5_ and NO_2_, whereas in our analyses HDL-C was positively associated with distance to roadways in our AA population. In a separate study of oxidized LDL in 79 diabetic individuals, a doubling in the distance to major roadways was associated with a decrease in oxidized LDL (Jacobs et al. 2011). Given the number of studies associating long-term air pollution exposure with lipid metabolism, further research should attempt to establish the specificity of these associations for specific sources and components of air pollution, as well as confirm their robustness using larger cohorts. In addition, more work should be done to establish the independence of, or a relationship between, associations of lipid metabolism and glucose control outcomes with long-term air pollution exposures.

*Strengths and limitations*. This study has several strengths and limitations. One limitation is that we used indirect measures of traffic-related air pollution, whereas alternative measures, such as black carbon, may more precisely estimate the exposure. In addition, because we used distance to roadways as our measure, we cannot discount the potential effect of traffic noise on our associations, particularly as other studies have noted an association between diabetes and traffic noise (Sørensen et al. 2013). Despite this limitation, distance to roadway has been shown to be highly correlated with particulate pollution from major roadways in North Carolina (Hagler et al. 2009). Another limitation is that the CATHGEN cohort is not a representative sample of the general population. As a cohort of outpatients undergoing outpatient cardiac catheterization procedures, the CATHGEN cohort has a CAD prevalence of 42%, which is higher than the estimated 6.7% national prevalence in 2006 (the midpoint year of the CATHGEN sample) (Centers for Disease Control and Prevention 2011). However, populations with CAD have been identified as particularly at-risk for complications resulting from air pollution exposure and thus are worthy of separate study (Brook et al. 2010).

The primary strength of this study comes from the depth of clinical information available in the CATHGEN cohort, allowing us to examine multiple metabolic outcomes while adjusting for relevant clinical and socioeconomic measures. In addition, all of these risk factors were assessed via physician-administered examinations or medical labs immediately before the catheterization procedure, reducing error in the measurements. An additional strength is the size of the CATHGEN cohort. Even after restricting to individuals residing in Durham, Wake, and Orange counties, there were sufficient observations to examine subcohorts and address race- and sex-specific associations. Our use of the TEZs allowed us to assess potentially traffic-pattern–specific sources of traffic-related air pollution and uncover associations that would have otherwise been missed.

## Conclusion

We have shown that distance to major roadways and residence in traffic exposure zones is associated with metabolic outcomes associated with CVD, including FPG and HDL-C. TEZs created to index both source and severity of TRAP on an ordinal scale added relevant information to associations observed, using distance to roadways as an index for TRAP. Associations with FPG were stronger in women, in line with previously published sex-specific associations, and we observed a novel association between distance to roadways and FPG among AA. Associations found in large retrospective cohorts such as CATHGEN lay the foundation for future exposure studies in prospective cohorts such as the Chinese Air Pollution and CardioMetabolic Disease cohort (Sun et al. 2013), as well as genetic and metabolomic studies to fully understand the impact of traffic-related air pollution.

## Supplemental Material

(303 KB) PDF
