# Supplementary material for: Association of Roadway Proximity with Fasting Plasma Glucose and Metabolic Risk Factors for Cardiovascular Disease in a Cross-Sectional Study of Cardiac Catheterization Patients
Source: Environ Health Perspect. 2015 Mar 24;123(10):1007–14. doi: 10.1289/ehp.1306980 (PMC4590740; doi:10.1289/ehp.1306980)

Environ Health Perspect
doi: 10.1289/ehp.1306980

__Note to Readers:__ ***EHP* strives to ensure that all journal content is accessible to all readers. However, some figures and Supplemental Material published in *EHP* articles may not conform to 508 standards due to the complexity of the information being presented. If you need assistance accessing journal content, please contact ehp508@niehs.nih.gov. Our staff will work with you to assess and meet your accessibility needs within 3 working days.**

Supplemental Material

Association of Roadway Proximity with Fasting Plasma Glucose and Metabolic Risk Factors for Cardiovascular Disease in a Cross-Sectional Study of Cardiac Catheterization Patients

Cavin K. Ward-Caviness, William E. Kraus, Colette Blach, Carol S. Haynes, Elaine Dowdy, Marie Lynn Miranda, Robert B. Devlin, David Diaz-Sanchez, Wayne E. Cascio, Shaibal Mukerjee, Casson Stallings, Luther A. Smith, Simon G. Gregory, Svati H. Shah, Elizabeth R. Hauser, and Lucas M. Neas

Table of Contents

Table S1. Spearman correlations for continuous outcomes.

Table S2. Distance to roadway associations.

Table S3. Traffic Exposure Zone Associations.

Figure S1. Association of FPG with distance to roadways before and after smoothing.
*Inter-quartile range scaling done after inverse-exponential transform. Dashed lines indicate the 95% confidence interval. Values on the y-axis are the estimated fasting plasma glucose (mg/dL) based on the non-linear model.

Table S1. Spearman correlations for continuous outcomes.

**Outcome**	**FPG**	**HOMA-IR**	**LDL-C**	**HDL-C**	**TC**	**TG**
FPG	1	0.53	-0.090	-0.14	-0.024	0.17
HOMA-IR		1	-0.020	-0.15	0.037	0.31
LDL-C			1	0.12	0.89	0.12
HDL-C				1	0.28	-0.35
TC					1	0.35
TG						1

Table S2. Distance to roadway associations.

Outcome	Subset	Beta (mg/dL) or Odds Ratio	CI (P)
		Beta (mg/dL)	
Fasting Plasma Glucose	Overall	2.17	-0.25, 4.58 (0.078)
	Men	0.14	-3.04, 3.33 (0.93)
	Women	5.16	1.48, 8.84 (0.006)
	EA	0.96	-1.61, 3.52 (0.47)
	AA	5.28	-0.17, 10.7 (0.058)
	Fasting Glucose ≥ 126	7.45	1.30, 13.6 (0.018)
		OR	
Diabetes (Fasting Glucose ≥ 126)	Overall	1.00	0.88, 1.13 (0.97)
	Men	0.92	0.78, 1.09 (0.35)
	Women	1.12	0.92, 1.36 (0.28)
	EA	1.00	0.86, 1.16 (1.00)
	AA	1.00	0.79, 1.26 (0.97)
		OR	
Diabetes (HP)	Overall	1.00	0.88, 1.13 (0.96)
	Men	0.97	0.83, 1.14 (0.71)
	Women	1.03	0.85, 1.26 (0.74)
	EA	0.98	0.84, 1.13 (0.75)
	AA	1.04	0.83, 1.31 (0.72)
		Beta	
HOMA-IR	Overall	-0.02	-0.53, 0.48 (0.93)
	Men	-0.12	-0.77, 0.52 (0.71)
	Women	0.08	-0.74, 0.89 (0.85)
	EA	0.15	-0.42, 0.72 (0.61)
	AA	-0.39	-1.37, 0.60 (0.44)
		Beta (mg/dL)	
HDL-C	Overall	-0.07	-1.14, 1.00 (0.89)
	Men	0.15	-1.07, 1.36 (0.81)
	Women	-0.58	-2.60, 1.43 (0.573)
	EA	-0.91	-2.13, 0.31 (0.15)
	AA	2.08	-0.09, 4.24 (0.06)
		Beta (mg/dL)	
LDL-C	Overall	-1.10	-4.12, 1.91 (0.47)
	Men	0.06	-3.64, 3.76 (0.98)
	Women	-2.84	-8.02, 2.35 (0.28)
	EA	-0.78	-4.23, 2.69 (0.66)
	AA	-2.10	-8.17, 3.98 (0.50)
		Beta (mg/dL)	
Triglycerides	Overall	-3.85	-14.6, 6.87 (0.48)
	Men	-8.65	-24.6, 7.26 (0.29)
	Women	3.90	-6.65, 14.5 (0.47)
	EA	-4.98	-19.2, 9.21 (0.49)
	AA	-1.12	-12.8, 10.6 (0.85)
		Beta (mg/dL)	
Total Cholesterol	Overall	-1.23	-4.46, 2.00 (0.46)
	Men	-0.59	-4.62, 3.44 (0.77)
	Women	-2.51	-7.95, 2.94 (0.37)
	EA	-1.49	-5.22, 2.24 (0.43)
	AA	-1.01	-7.46, 5.44 (0.76)

Outcome	Subset	Beta (mg/dL) or Odds Ratio	CI (P)
		OR	
Hyperlipidemia	Overall	1.09	0.96, 1.23 (0.17)
	Men	1.01	0.86, 1.18 (0.94)
	Women	1.23	1.01, 1.50 (0.042)
	EA	1.05	0.91, 1.21 (0.31)
	AA	1.20	0.95, 1.51 (0.13)

Distance to roadway associations with all outcomes. The beta coefficients (Beta) are from linear regression analysis of continuous dependent variables and OR from logistic regression analysis of binary outcomes with 95% confidence intervals (CI) and p-values (P)) overall and for gender and race stratified models. Models were adjusted for race, sex, socio-economic status, BMI, and smoking status. For the Men/Women stratifications the sex term was dropped from the model and for the EA/AA associations the race term was dropped from the model.

Table S3. Traffic Exposure Zone Associations.

Outcome	Exposure Zone	Beta (mg/dL) or Odds Ratio	CI (P)
Fasting Plasma Glucose	TEZ 2	-1.36	-7.20, 4.48 (0.65)
	TEZ 3	1.26	-5.84, 8.34 (0.73)
	TEZ 4	4.12	-2.23, 10.5 (0.20)
	TEZ 5	6.42	-11.3, 24.2 (0.48)
	TEZ 6	7.57	-14.6, 29.7 (0.50)
	TEZ 5/6	13.2	-22.7, 49.1 (0.47)
	TEZ Trend	1.72	-0.125, 3.57 (0.068)
	TEZ Trend (Fasting Glucose ≥ 126)	4.80	0.097, 9.51 (0.046)
		OR	
Diabetes (Fasting Glucose ≥ 126)	TEZ 2	0.96	0.70, 1.31 (0.79)
	TEZ 3	0.89	0.61, 1.30 (0.54)
	TEZ 4	1.02	0.73, 1.42 (0.91)
	TEZ 5	0.89	0.35, 2.29 (0.82)
	TEZ 6	1.30	0.43, 3.94 (0.64)
	TEZ 5/6	1.04	0.49, 2.17 (0.93)
	TEZ Trend	1.00	0.92, 1.11 (0.86)
		OR	
Diabetes (HP)	TEZ 2	0.98	0.73, 1.31 (0.88)
	TEZ 3	0.78	0.54, 1.13 (0.19)
	TEZ 4	0.95	0.69, 1.30 (0.73)
	TEZ 5	0.69	0.28, 1.75 (0.44)
	TEZ 6	0.94	0.30, 2.98 (0.91)
	TEZ 5/6	0.77	0.37, 1.63 (0.50)
	TEZ Trend	0.97	0.88, 1.06 (0.47)
		Beta	
HOMA-IR	TEZ 2	-1.45	-2.91, 0.01 (0.05)
	TEZ 3	-1.18	-2.89, 0.53 (0.18)
	TEZ 4	-0.20	-1.76, 1.37 (0.81)
	TEZ 5	-3.29	-7.76, 1.18 (0.15)
	TEZ 6	-2.75	-7.89, 2.39 (0.29)
	TEZ 5/6	-3.06	-6.51, 0.39 (0.08)
	TEZ Trend	-0.17	-0.53, 0.20 (0.37)
		Beta (mg/dL)	
HDL	TEZ 2	0.84	-1.88, 3.56 (0.55)
	TEZ 3	1.29	-2.04, 4.62 (0.45)
	TEZ 4	1.32	-1.56, 4.20 (0.37)
	TEZ 5	8.36	-0.15, 16.9 (0.054)
	TEZ 6	5.98	-3.96, 15.9 (0.24)
	TEZ 5/6	7.36	0.74, 14.0 (0.03)
	TEZ TREND	0.65	-0.18, 1.48 (0.13)
		Beta (mg/dL)	
LDL	TEZ 2	-1.27	-9.09, 6.55 (0.75)
	TEZ 3	-0.19	-9.71, 9.34 (0.97)
	TEZ 4	2.70	-5.57, -10.8 (0.52)
	TEZ 5	-17.2	-44.3, 9.85 (0.21)
	TEZ 6	2.75	-22.9, 28.4 (0.83)
	TEZ 5/6	6.69	-25.8, 12.4 (0.49)
	TEZ TREND	0.73	-1.62, 3.08 (0.54)
		Beta (mg/dL)	

Outcome	Exposure Zone	Beta (mg/dL) or Odds Ratio	CI (P)
Triglycerides	TEZ 2	-13.10	-40.34, 14.1 (0.34)
	TEZ 3	1.00	-32.3, 34.3 (0.95)
	TEZ 4	-9.60	-38.4, 19.2 (0.51)
	TEZ 5	-7.66	-92.5, 77.2 (0.86)
	TEZ 6	-41.30	-140, 57.9 (0.42)
	TEZ 5/6	21.7	-87.9, 44.5 (0.52)
	TEZ TREND	-1.95	-10.3, 6.37 (0.65)
		Beta (mg/dL)	
Total Cholesterol	TEZ 2	-3.97	-12.2, 4.26 (0.34)
	TEZ 3	-2.04	-12.1, 8.05 (0.69)
	TEZ 4	-1.08	-9.82, 7.66 (0.81)
	TEZ 5	-4.12	-29.8, 21.6 (0.75)
	TEZ 6	0.36	-29.6, 30.4 (0.98)
	TEZ 5/6	-2.24	-22.2, 17.8 (0.83)
	TEZ TREND	0.19	-2.40, 2.63 (0.93)
		OR	
Hyperlipidemia	TEZ 2	0.94	0.70, 1.26 (0.69)
	TEZ 3	0.98	0.69, 1.40 (0.91)
	TEZ 4	0.88	0.64, 1.21 (0.44)
	TEZ 5	1.32	0.53, 3.28 (0.56)
	TEZ 6	1.29	0.41, 4.11 (0.67)
	TEZ 5/6		
	TEZ TREND	0.99	0.90, 1.08 (0.76)

Associations for each of the traffic exposure zones with each outcome. For the TEZ associations, in addition to the association with each of the TEZs (TEZ 1 taken as the baseline for comparisons) the association with an ordinal variable encoding each of the TEZs is given (TEZ Trend). Results are given as Beta coefficients from linear regression models for continuous variables or OR from logistic regression models along with 95% confidence intervals (CI) and p-values (P). TEZ 5/6 refers to associations when TEZ 5 and TEZ 6 were combined. Models were adjusted for race, sex, socio-economic status, BMI, and smoking status.

The TEZ trend p-values were derived by using an ordinal variable across the traffic exposure zones in a linear regression model. All covariate adjustments remained the same.

Figure S1. Association of FPG with distance to roadways before and after smoothing.
*Inter-quartile range scaling done after inverse-exponential transform. Dashed lines indicate the 95% confidence interval. Values on the y-axis are the estimated fasting plasma glucose (mg/dL) based on the non-linear model.

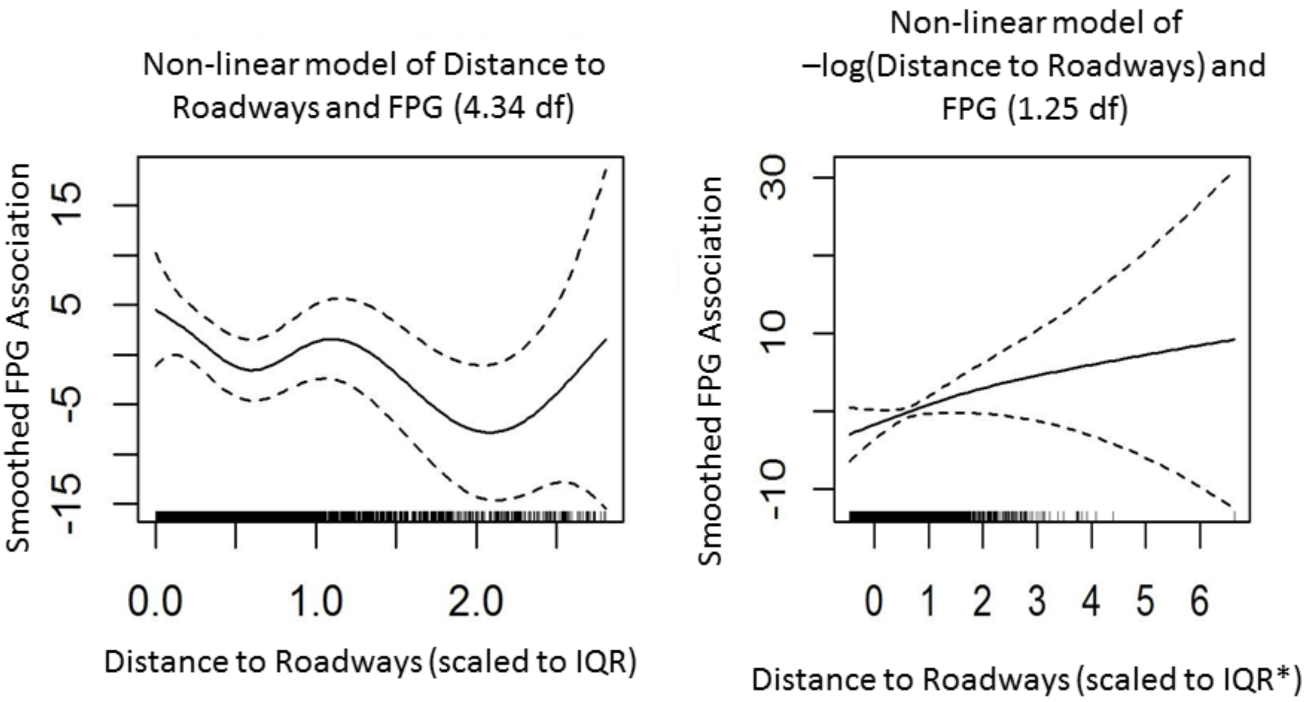

Supplement: (303 KB) PDF [file ehp.1306980.s001.acco.pdf]
